# Facile fabrication of solution-processed solid-electrolytes for high-energy-density all-solid-state-batteries by enhanced interfacial contact

**DOI:** 10.1038/s41598-020-68885-4

**Published:** 2020-07-17

**Authors:** Min-Ju Kim, Jun-Woo Park, Byung Gon Kim, You-Jin Lee, Yoon-Cheol Ha, Sang-Min Lee, Kang-Jun Baeg

**Affiliations:** 1 Next Generation Battery Research Center, Korea Electrotechnology Research Institute, Changwon, Gyeongsangnam-do 51543 Republic of Korea; 2 Department of Graphic Arts Information Engineering, Pukyong National University, Busan, 48513 Republic of Korea

**Keywords:** Energy science and technology, Materials science

## Abstract

Instead of commercial lithium-ion batteries (LIBs) using organic liquid electrolytes, all-solid-state lithium-ion batteries (ASSBs) employing solid electrolytes (SEs) are promising for applications in high-energy–density power applications and electric vehicles due to their potential for improving safety and achieving high capacity. Although remarkable progress in SEs has been achieved and has resulted in high ionic conductivity, which now reaches values comparable to those of liquid electrolytes, the typical use of a slurry process for the fabrication of conventional ASSBs inevitably causes harmful reactions between sulfide materials and polar solvents. Here, we studied the efficient infiltration process of SE slurry into conventional composite LIB electrodes (NCM622) for achieving high-energy-density ASSBs via a scalable solution-based fabrication process. Two methods are proposed to ensure that SE materials are evenly distributed and sufficiently infiltrated into the porous structures of LIB electrodes. The LPSCl SE solutions were effectively infiltrated into the electrodes at higher processing temperatures and the temperature was subsequently optimized at above the boiling point of the ethanol solvent due to the dynamic motion of SE molecules via a convective flow during solvent vaporization. Moreover, the porous LIB composite electrodes with a mixture of active materials of different particle sizes formed and filled capillary pores resulting in a high electrode density. The LPSCl SE-infiltrated NCM622 electrodes that used this strategy could remarkably improve the initial discharge capacity of ASSBs to as high as 177 mAh/g. These ASSBs also showed excellent performance even at high loading values (about 17 mg/cm^2^), making them competitive with LIBs using conventional liquid electrolytes.

## Introduction

Recently, the demand for rechargeable energy storage devices has increased remarkably, especially for electric vehicles, mobile/wearable smart devices, and electrical energy storage (EES) systems^1^. Lithium ion batteries (LIBs) have been widely used in most portable electronic applications, such as smart phones and laptop computers, due to their high energy and power densities^2,3^. Conventional LIBs use liquid electrolytes (LEs) which are manufactured by dissolving lithium salts in organic solvents as their main elements^4^. However, the LEs commonly used in LIBs are intrinsically dangerous, as they cause too many safety issues and are difficult manufacture on large scale due to their volatility and flammability especially at high operating temperatures. In contrast, all-solid-state batteries (ASSBs), which have the same structure as conventional LIBs but with solid electrolytes (SEs) instead of LEs, have attracted tremendous interest as a promising technology to achieve high energy and power capacity as well as stable operation even under high temperature conditions without the risk of ignition or explosion, because SEs are basically nonflammable^5–7^. Moreover, SEs also have a variety of other advantages in terms of their compatibility with Li metal as an ideal high-energy–density anode. SEs exhibit superior electrochemical oxidation potentials (stable above 5.0 V vs Li/Li^+^) in comparison to LEs (oxidation reaction around 4.0 V vs Li/Li^+^), which leads to better material compatibility with higher potential electrodes for increased energy density. Adopting SEs makes it possible to reduce the weight of inactive components, such as packaging materials and thermal managing elements in batteries, which could provide more freedom for cell design via stacking of bipolar electrodes^8^.

Inorganic SEs in ASSBs have the same operating principle as conventional organic LEs, and many types of SEs have been developed in the past several years, including garnet-type, NASICON-type, perovskite-type, LISICON-type, sulfide-type, and argyrodite-type SEs^9^. Among those various types of SEs, sulfide-type, and argyrodite-type SEs are very promising in terms of their large scale solution processability, deformable mechanical properties, high ionic conductivities, and excellent electrochemical performance^10^. Sulfide materials are deformable because two-dimensional ionic contacts with active materials can be formed by a simple mechanical cold-press process. Moreover, since the interaction of S^2−^ with Li^+^ ions is weaker than that between O^2−^ and Li^+^, most sulfide-type SEs have shown higher ionic conductivities and Li^+^ mobilities than oxide-type SEs. Therefore, recent progress in SEs based on sulfides has resulted in the highest ionic conductivity at room temperature yet reported at over 10^–2^ S cm^−1^ (e.g., Li_10_GeP_2_S_12_, and Li_7_P_3_S_11_); those values are now comparable to the ionic conductivities of organic LEs^11–16^. Argyrodite-type Li-ion SEs, such as Li_6_PS_5_X (X = Cl, Br, I), also have high ionic conductivities similar to sulfide-type SEs. After high-energy ball-milling and post-annealing processes, Li_6_PS_5_Cl (referred to here as LPSCl) readily showed ionic conductivities above 10^–3^ S cm^−1^ at room temperature. The high Li-ion diffusion pathways with low activation energy contribute to long-range transport in the fully disordered argyrodite structure of LPSCl; thus, the disorder between the S^2−^ and Cl^-^ ions promotes Li-ion mobility.

Although sulfide- and argyrodite-type SEs exhibit the highest ionic conductivities, they still have to overcome several issues for successful application in commercial ASSBs. First, they are not stable in ambient air due to their reaction with moisture. Using these materials requires a controlled environment and encapsulation. In addition, scalable deposition techniques are needed for the production of sulfide-type SEs. ASSBs employing sulfide SE materials have been developed using the conventional LIB process. However, the process is not scalable since the bulk-type ASSB electrodes were mostly fabricated using a dry process, that is, mixing of active materials, SEs, and carbon additives, followed by a mechanical pressing process. In view of commercialization, however, electrodes fabricated by such dry process have many limitations, in terms of the large-scale electrodes needed for low-cost battery fabrication, as well as the poor mechanical properties. Therefore, ASSBs based on sulfide-type SEs require scalable sheet-type electrodes fabricated by wet/solution processes with polymeric binders, such as poly(vinylidene fluoride) (PVDF), poly(vinyl alcohol) (PVA), or carboxymethyl cellulose (CMC), and subsequently require the homogenization of active materials with conducting additives, binders, and SEs. To this end, a suitable combination of polymeric binders and solvents is vitally important to obtain proper conduction pathways for Li ions and electrons simultaneously. However, a slurry process for ASSB electrodes can be employed with a limited library of polymeric binders and their proper solvents. Notably, for dissolving PVDF, PVA, and CMC binders, polar solvents, such as N-methyl-2-pyrrolidinone (NMP) and water, are typically used, but those polar solvents are critically reactive with most sulfide materials^17^. In contrast to LEs, therefore, it is difficult to form the optimum combination of active materials and sulfide-type SEs. This poor ionic and electrical contact inevitably causes large interfacial resistance.

For commercial application of SEs and their electrodes for high capacity ASSBs, a wet/solution process using a slurry coating method is preferable. Recently, 80 × 60 mm^2^ ASSB electrodes were fabricated by using a liquefied SE, where the sheet-type electrodes were readily prepared by infiltration of SE solutions (LPSCl in ethanol (EtOH) or 0.4LiI-0.6Li_4_SnS_4_ in methanol) into conventional LIB composite electrodes with a PVDF binder^18^. Because of superior ionic contacts and percolating conductive pathways, the composite electrodes showed relatively high cell-based energy capacities of 141 and 364 mA h g^−1^ using LiCoO_2_ and graphite electrodes, respectively. In spite of this progress, the cell-based energy density is still not competitive with that of conventional LIBs. This shortcoming is attributed to the relatively low density of the electrodes due to insufficient infiltration of SEs into the electrodes. Thus, the performance of ASSB electrodes manufactured by the wet-process still have a long way yet to go before commercialization.

Here we report an efficient infiltration process of solution-based LPSCl SEs into NCM622-based composite cathodes for high capacity ASSBs. To facilitate the infiltration of more SE into the electrodes, dynamic molecular motion of SEs is induced at elevated process temperature, even by increasing the temperature above the boiling point of the solvents. The electrochemical performance of ASSBs employing the LPSCl-infiltrated SEs is strongly dependent on the LPSCl content, and it is appreciably increased at higher temperature. The initial discharge capacities of ASSBs based on SE-infiltrated NCM622 and Li-In half cells increased remarkably from 72 mA h g^−1^ to 136 mA h g^−1^ at 45 °C and 90 °C at high loading value (15.3 mg/cm^2^ and 17 mg/cm^2^), respectively. Vaporization of solvents contributes to the deep penetration of SE ions and their uniform distribution over the active materials in the cathodes of ASSBs. Moreover, active materials with various particle sizes are investigated to study their effect on SE solution infiltration. In contrast to single-size active materials, mixed small- (4 µm) and large- (10 µm) size active particles lead to better infiltration of SEs and superior electrical contact between the LPSCl SEs and cathodes. Therefore, a significantly higher discharge capacity is obtained by using a mixture of differently sized active materials, mainly due to the capillary phenomena in porous electrodes which absorb the SE solution, thereby providing improved ionic transport pathways.

## Results and discussion

**Figure 1:**
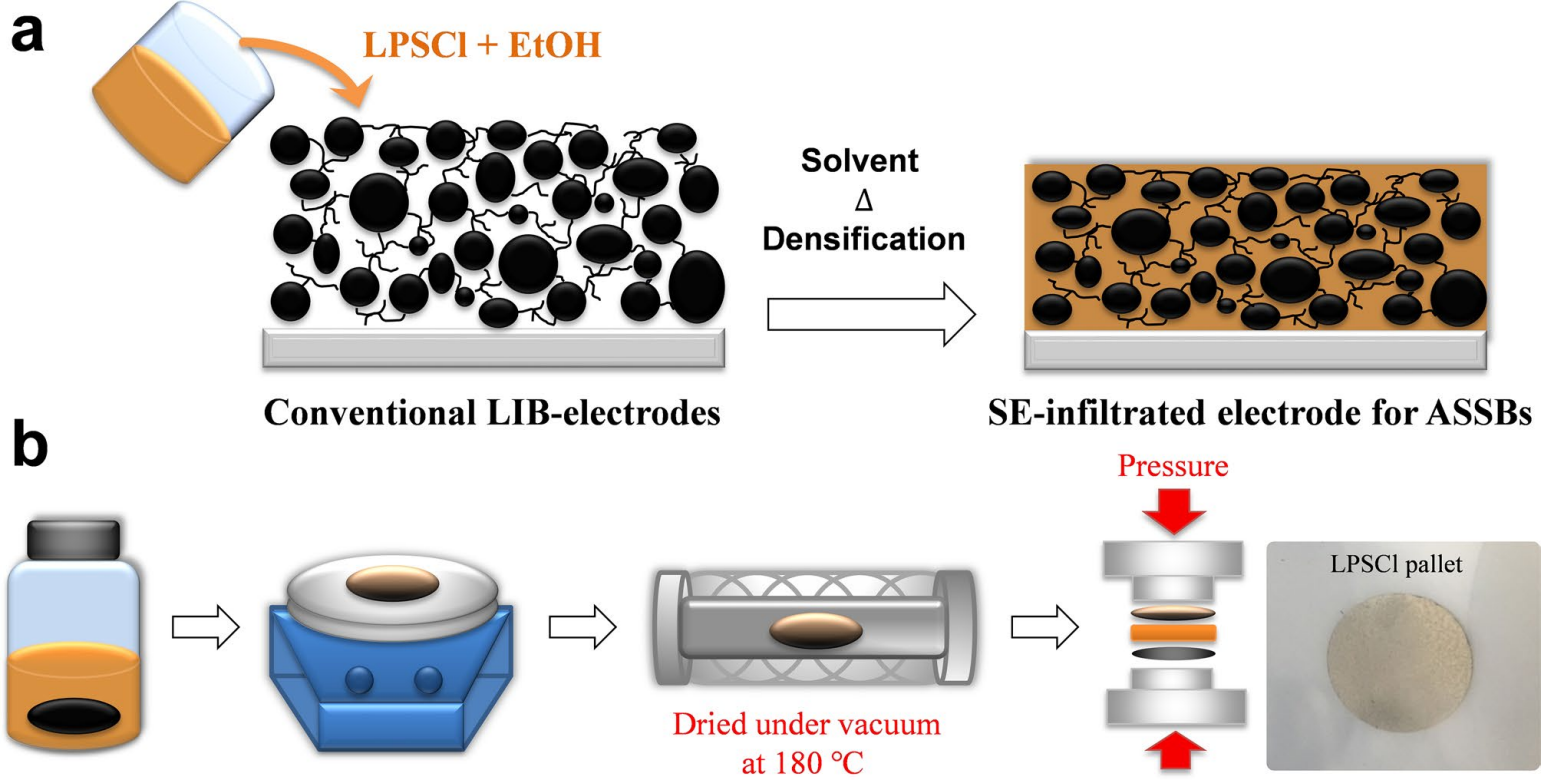
(**a**) Schematic of the solution-based infiltration process of a solid-electrolyte (LPSCl in EtOH) into conventional LIB electrodes (NCM622-based composite cathode), (**b**) and the fabrication steps: dipping in electrolyte solution, drying on a hotplate, thermal annealing under vacuum conditions, and cold pressing to assemble the electrode components of all-solid-state batteries.

Although the layered transition metal oxide LiCoO_2_ has a high Li^+^ ion conductivity and theoretical specific capacity of 272 mA h g^−1^^19^, cathodes based on it showed a low practical capacity of ~ 140 mA h g^−1^, mainly due to modifications in its structure during charge and discharge cycles. As an alternative to LiCoO_2_, we used commercial NCM622 cathodes, consisting of a co-doped LiCoO_2_ system with Co and Mn, to ensure higher capacity and stable operation during cycling. The commercial NCM622 (active materials) was blended into NMP solvent with PVDF polymeric binders and carbon black in order to obtain simultaneously good conductors both for ions and electrons. The composite cathode layer, that is, the conventional LIB-electrodes shown in Fig. 1a, was formed by casting a wet-slurry onto a current collector (Al foil) followed by thermal baking at 100 °C to remove residual NMP solvent. For low-cost and large-scale fabrication of SE-infiltrated NCM622 composite cathodes, a sulfide-type LPSCl SE solution (42.8:41.4:15.8 wt.% of Li_2_S/P_2_S_5_/LiCl) was prepared by a wet chemical synthesis using SE precursors such as Li_2_S, P_2_S_5_, and LiCl, in EtOH solvent (LPSCl + EtOH solution in Fig. 1a). Figure 1b shows the infiltration process of LPSCl SE solution into the conventional LIB electrodes. The LIB electrodes were dipped in the as-prepared LPSCl solution, and then samples were dried in an oven followed by thermal treatment at 180 °C under vacuum in order to solidify the SE films as well as fully remove the residual solvent. The fabrication of SE-infiltrated NCM622 electrodes was completed by pressing the samples under load of 700 MPa to induce intimate contact between active materials and the SE and better connection by increasing the density of the films (low porosity). LPSCl-infiltrated LIB electrodes typically exhibits a yellow color due to the existence of sulfide-type SEs, as can be seen in the inset image of Fig. 1b. Notably, this solution-based SE-infiltration process could be further modified by using various roll-to-roll and/or large-area graphic arts printing techniques, such as spray, gravure, and screen printing, for large-scale cost-effective fabrication of ASSBs^20^.

**Figure 2:**
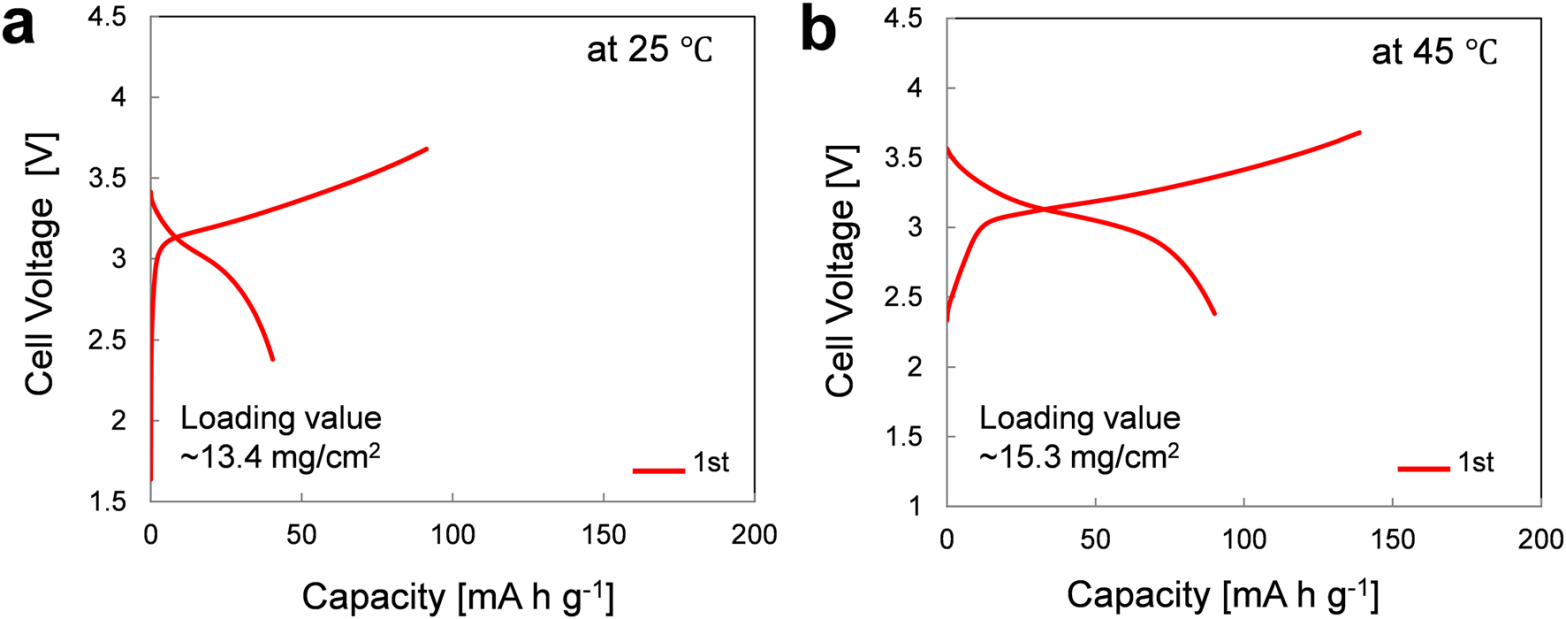
Electrochemical characterization of all-solid-state NCM622/Li-In half-cells after infiltration of the LPSCl SE into the cathode at various preparation temperatures. First-cycle charge and discharge voltage profiles of the SE-NCM electrode (**a**) at a loading value of 13.4 mg/cm^2^ infiltrated at room temperature (25 °C) and (**b**) at a loading value of ~ 15.3 mg/cm^2^ infiltrated at elevated temperature (45 °C).

Electrochemical characterization of the LPSCl SE-infiltrated NCM622-based composite electrodes (below refer to SE-NCM) was carried out using SE-NCM/Li-In all-solid-state half-cells. Figure 2 shows the voltage profiles during initial charge and discharge cycles at 0.05 C. To investigate the efficiency of the solution infiltration process in SE-NCM, two samples were prepared by dipping into the SE solution at different solution temperatures. Obviously, the loading concentration of the LPSCl SE solution increased from 13.4 to 15.3 mg/cm^2^ when the SE-NCM electrodes were infiltrated at 25 °C and 45 °C, respectively. At room temperature, SE-NCM/Li-In cells showed a very low reversible capacity of 40 mA h g^−1^. On the other hand, when the solution temperature was increased to 45 °C in order to induce more efficient penetration of SE into the composite electrodes, we obtained a higher reversible capacity of 72 mA h g^−1^ compared the electrodes infiltrated at 25 °C. Although the capacities of the all-solid-state cells were significantly lower than those of a LE cell, the loading value of the SE-NCM composite electrodes was increased at higher temperature due to better infiltration of the SE solution so that the cells showed higher capacity at elevated temperature.

**Figure 3:**
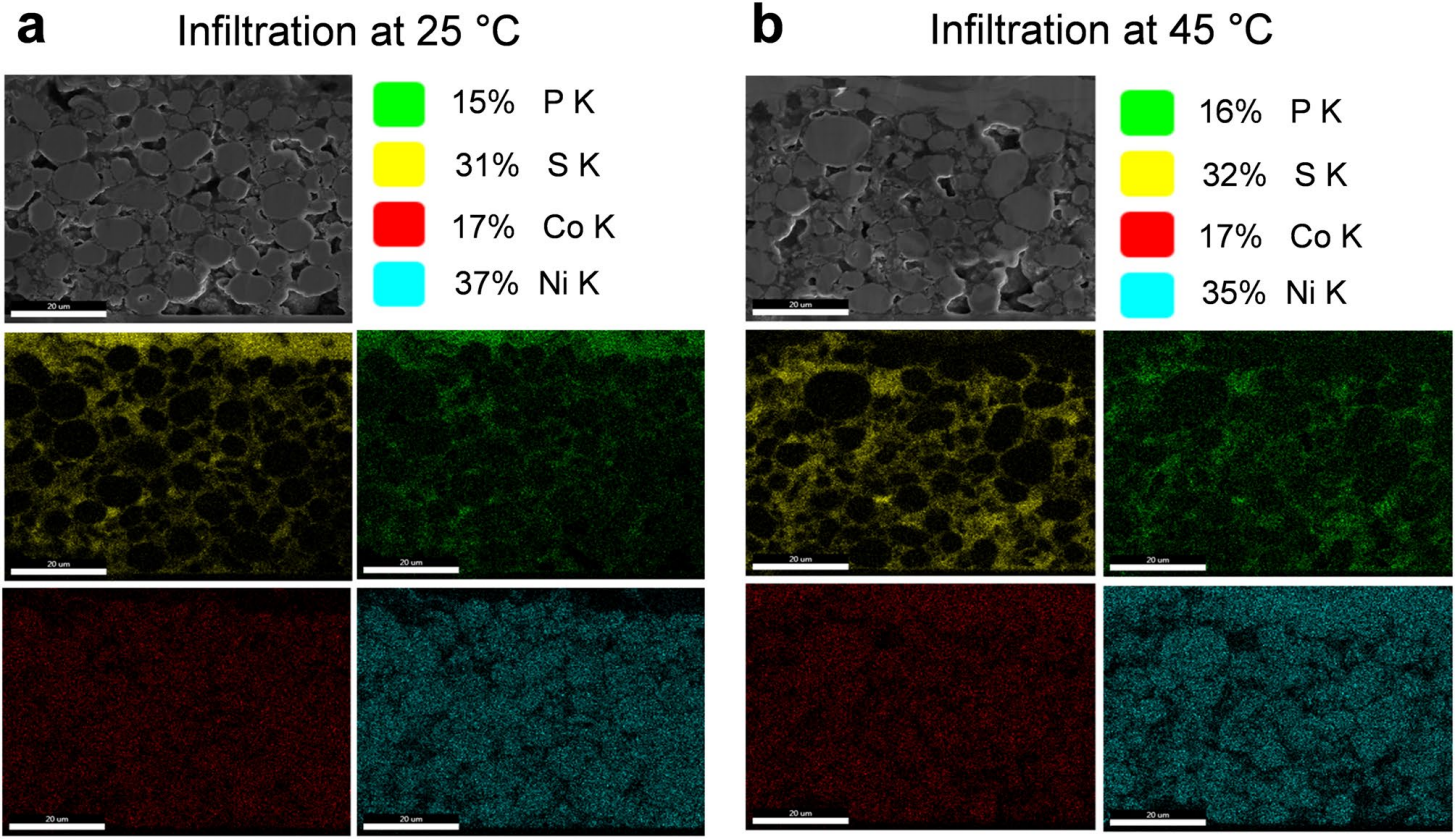
Cross-sectional FE-SEM images of LPSCl-infiltrated NCM622-based composite electrodes and their corresponding EDXS elemental maps. The cathodes were prepared by the solid electrolyte (SE) infiltration process at different temperatures: (**a**) at room temperature and (**b**) at elevated temperature (45 °C). The loading value of the electrodes was ~ 17.92 mg/cm^2^. After cutting the electrode cross-section using a FIB, the spatial distributions of SE elements (representatively S ions) were analysed and the uniform distribution of SE inside the electrodes was confirmed when processed at 45 °C.

Figure 3a,b show cross-section FESEM images and their corresponding elemental maps obtained by energy dispersive X-ray spectroscopy (EDXS) for the SE-NCM electrodes prepared at 25 °C and 45 °C, respectively. After cutting and polishing the cross-sections of electrodes via FIB milling, spatial distribution maps were captured by measuring the elements present in the NCM622 active materials, such as Co (red color) and Ni (blue color), which showed uniform distributions in all samples. To confirm the SE infiltration, representative S (yellow color) and P (green color) elements from the LPSCl were analyzed using the same EDXS method. Notably, SE-NCM electrodes that prepared by SE solution infiltration at 45 °C showed very uniform distributions of those elements, even deep inside the composite electrodes, while those electrodes fabricated at room temperature had a high concentration of S and P elements on the top surface of the electrodes. This reveals that the LPSCl SE solution could effectively penetrate more deeply into the LIB electrodes at higher processing temperature. This higher penetration efficiency was mainly achieved by the higher diffusion coefficient of ions at higher temperature and reveals the excellent deformability of the LPSCl SEs. Moreover, this solution/wet process could enable the formation of intimate ionic contacts between electrodes and electrolytes as well as densify the cathodes to achieve high specific capacity all-solid-state LIBs.

**Figure 4:**
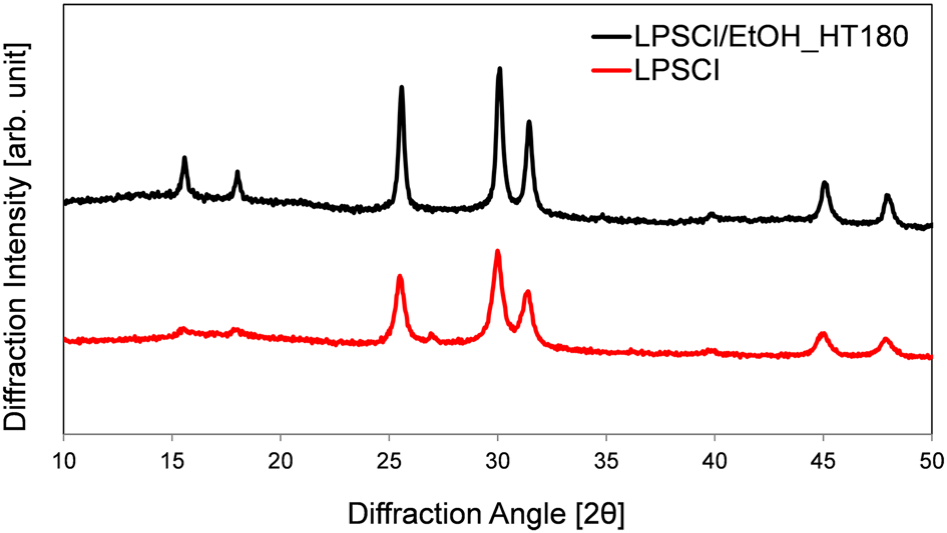
Crystallographic characterization of the LPSCl solid electrolyte. XRD patterns for pristine LPSCl (red line) and LPSCl after evaporating the solvent on a hotplate followed by thermal treatment at 180 °C under vacuum conditions (black line).

For crystallographic analysis of the LPSCl SE materials, XRD patterns were obtained for as-prepared LPSCl (red line) and for samples formed after solution-casting and heat treatment at 180 °C under vacuum (black line), as can be seen in Fig. 4. Both pristine and thermal-treated LPSCl SEs had similar peaks at the same positions. Those characteristic diffraction peaks are consistent with the positions of peaks indexed to the crystallized argyrodite Li_6_PS_5_Cl phase^21^, which may be spontaneously formed during the preparation steps (high-energy ball-milling and thermal treatment)^22^. It is also reported that the argyrodite phase of Li_7_PS_6_ is easily formed between 80 and 150 °C during the heating of the ball-milled glass ceramic phase of LPSCl SEs. Under higher temperature thermal treatment conditions, Cl was incorporated into the Li_7_PS_6_ so that it formed a crystalline Li_7−x_PS_6−x_Cl_x_ phase^23^. Moreover, there were no impurity peaks in the XRD patterns, which confirmed the formation of a pure crystallized Li_6_PS_5_Cl phase. This suggests that during the solidification of the LPSCl SE solution, there was no influence on the presence of other components of the composite electrodes, and the active materials (NCM622) also remained intact in contact with the SE-dissolved EtOH solutions.

**Figure 5:**
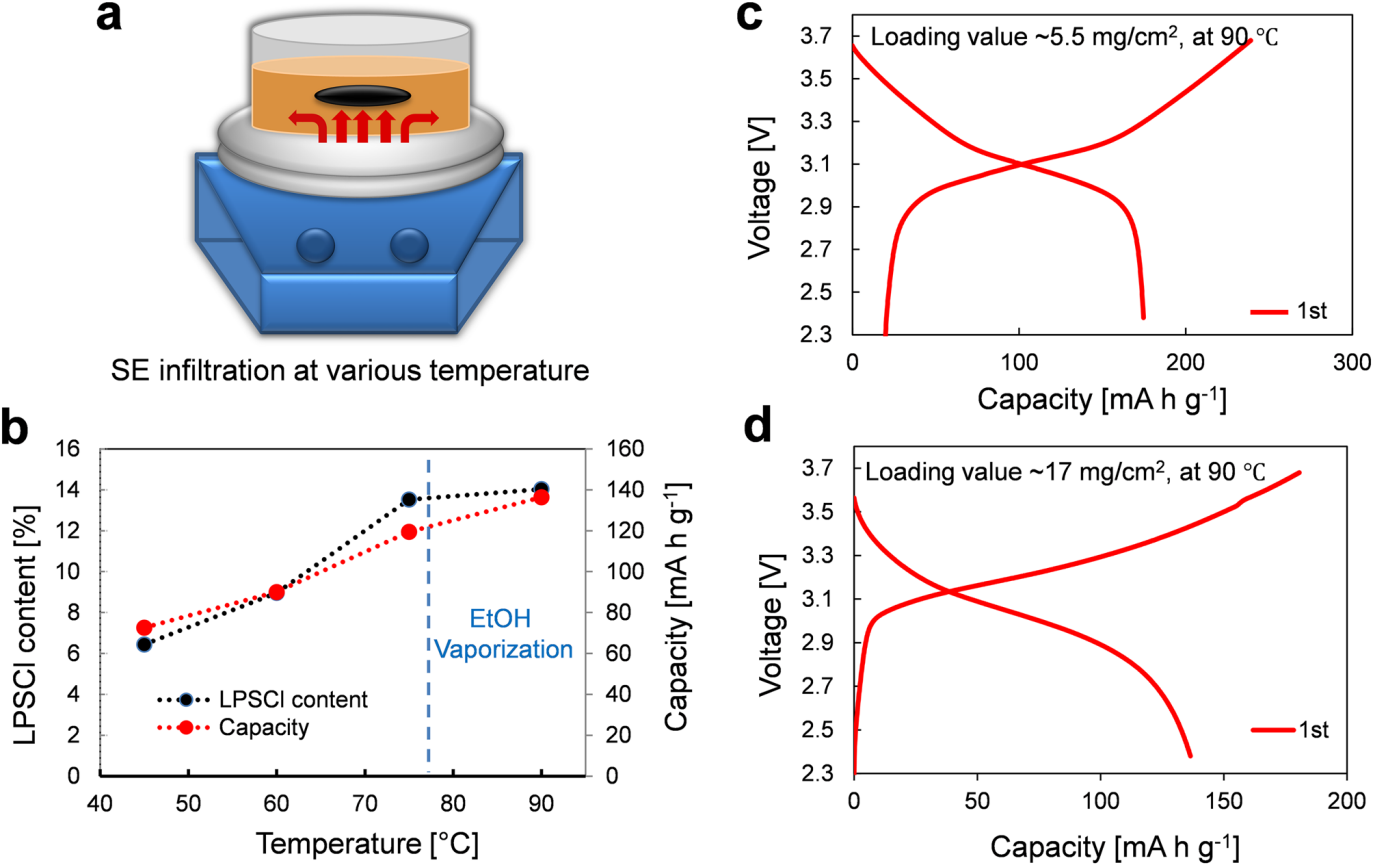
Effects of solvent vaporization on the LPSCl SE-infiltration process in the cathodes of all-solid-state batteries. (**a**) Schematic of SE-solution infiltration process at various temperatures from 45 to 90 °C on a temperature-controlled hotplate, and (**b**) corresponding LPSCl contents and initial discharge capacities of the SE-NCM/Li-In half cells. Considering that the boiling point of EtOH solvent is 78.3 °C (dotted line), the vaporization effect begins to contribute around this point, so that a uniform distribution of SE is induced in the electrodes by convective flow. First-cycle charge and discharge voltage profiles of the same half cells employing the SE-infiltrated cathodes processed above the boiling point of EtOH (at 90 °C) with a loading value of (**c**) ~ 5.5 mg/cm^2^ and (**d**) ~ 17 mg/cm^2^.

The electrochemical characteristics of the wet-processed ASSBs are intimately affected by the SE content infiltrated into the LIB electrodes. Although the above LPSCl SE solution was confirmed to more effectively infiltrate into the NCM622-based composite electrodes at 45 °C, the capacity was still low in comparison to conventional LIBs based on LEs (~ 154 mA h g^−1^). In order to achieve higher SE content by more effective penetration of the SE elements, we further increased the temperature. Figure 5a,b show schematic images of SE-infiltration process at various bath temperatures from 45 to 90 °C, and the corresponding amounts of LPSCl SE in the cathodes and the resulting reversible capacity of all-solid-state LIBs, respectively. After dipping the NCM622 electrodes in the SE-solution, the hotplate temperature was slowly raised up to 90 °C. Because the EtOH in the SE solution has a relatively low boiling point at 78.3 °C, the temperature is limited near the boiling point of the solvents. As the process temperature was increased, the SE content also increased from 6.4 to 14%. Because we controlled the loading value of conventional LIB electrodes at ~ 18 mg/cm^2^, the higher concentration of SEs simply leads to a higher density of electrodes by filling the pores. The initial discharge capacity of all-solid-state LIB half-cells remarkably improved from 72 to 136 mA h g^−1^ when the temperature was increased from 45 to 90 °C, respectively. The specific capacity is strongly related to the amount of SE infiltration. This was mainly attributed to the fact that the SE ionic elements could become more active at higher temperature due to increased dynamic molecular motion and enable further infiltration deeper into the composite electrodes. Furthermore, when the temperature of the SE-solution is increased above the boiling point of the solvent at 90 °C, solvent vaporization could play an important role for efficient penetration of SEs via significantly enhanced molecular motion of ionic elements in the convective flow of the SE solution. As the EtOH solvent evaporates, air gaps present in the LIB electrodes can be released so that the SE can fill those spaces. Therefore, we achieved a high capacity of all-solid-state LIBs based on SE-NCM and Li-In half-cells of about 177 mA h g^−1^ with a loading value of 5.5 mg/cm^2^ (see Fig. 5c). This capacity is higher than those of commercial LIBs with LEs is approaching the theoretical capacity of NCM622 cathode-based ASSBs^24^. Consequently, our SE-NCM cathodes wet-processed above the solvent evaporation temperature (90 °C) outperformed the same electrodes that were prepared at a lower temperature. It is also worth noting that they still showed a good capacity of 136 mA h g^−1^, even with loading values three-fold higher at ~ 17 mg/cm^2^ in comparison to the low loading of 5.5 mg/cm^2^ (see Fig. 5d). In addition, the capacity and the efficiency of the first charge cycles are higher than that of the second discharge cycles due to the reaction of the lithium-ion metal oxides and the sulfides solid electrolytes during the initial charge process. Therefore, lithium is used to form the surface layer during the initial charge cycle, which results in an irreversible reaction^25^.

The long duration of the cycling test is required to verify the reliability of SE-infiltrated composite electrodes in an ASSB cell. We obtained the electrochemical characteristics during 20 cycles of the charge–discharge processes, which exhibited relatively reliable and reproducible results. Fig. S1a in the supporting information shows charge and discharge voltage profiles and their capacities of ASSBs using the SE-infiltrated NCM622-based composite cathodes at work temperature of 55 °C, C-rate of 0.05 C, and a loading value of 2.3 mg/cm^2^. We obtained high capacities of 238.09 and 172.58 mA h g^−1^ during the initial charging and discharging processes, respectively. It is noted that the initial capacity did not significantly change after 20 cycles. When we measure the Coulombic efficiency of the same cells, it maintains 97% efficiency after finishing 20 cycles (see Fig. S3). Moreover, Fig. S1b shows the charge transfer resistance of SE-infiltrated NCM622-based composite electrodes before and after the duration test. It could conclude that SEs-infiltrated cathode was successfully transferred ions even after 20 charge–discharge cycles since there is no significant change in the internal resistance of a cell. Moreover, the cross-sectional FE-SEM images and their EDXS elemental mapping analysis were also conducted to investigate that the SEs distribution and possibility of secondary phase formation after cycling tests. Fig. S2 exhibits a spatial distribution of P, S, Co, and Ni elements, where the LPSCl SEs uniformly coated and there was no evidence to form the secondary phases in the cathodes after 20 cycling tests.

**Figure 6:**
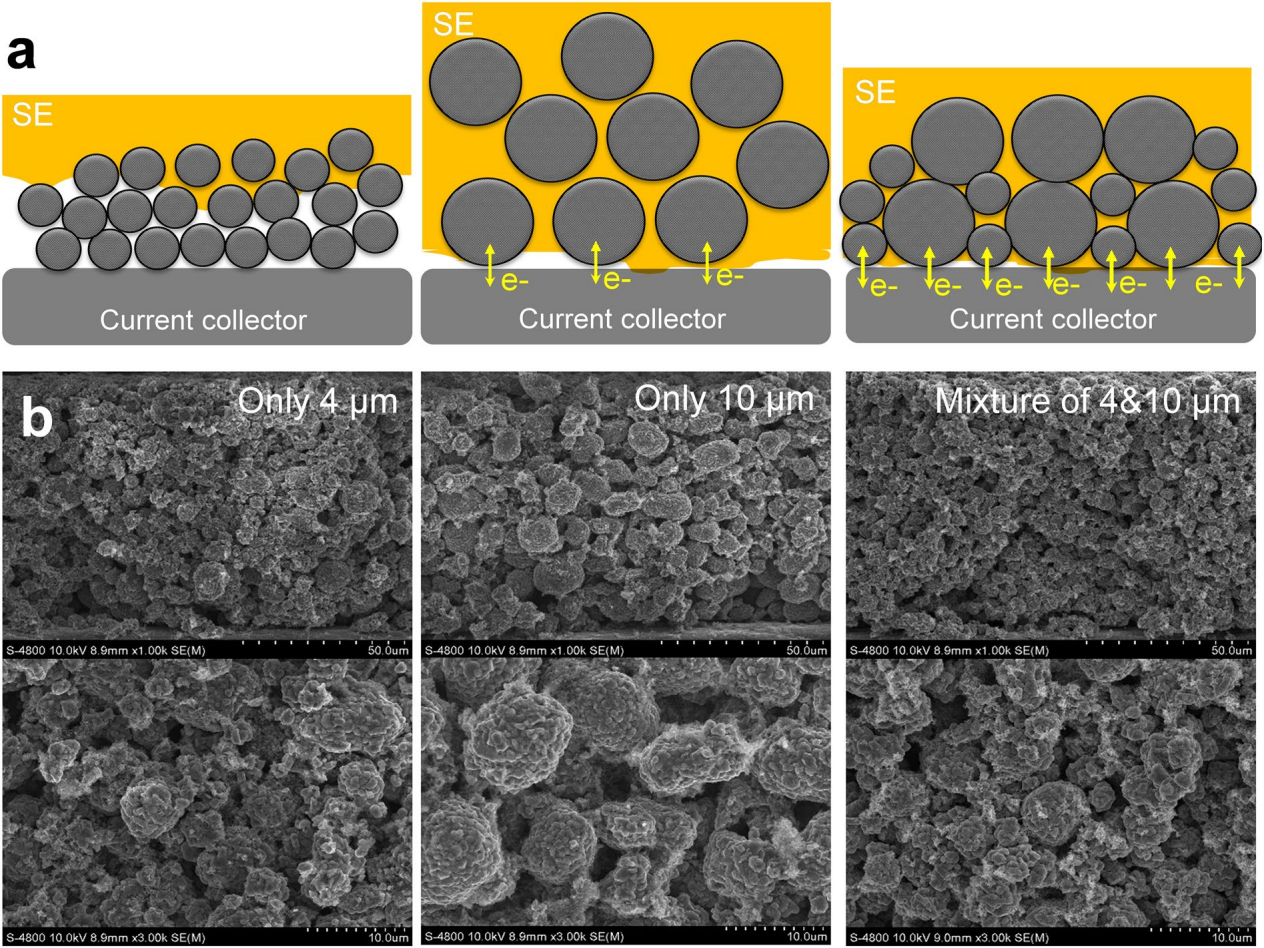
Comparison of LPSCl distribution inside the electrodes using different sizes of active materials: either small (only 4 µm) and large (only 10 µm) particles or a mixture of two different sized particles. (**a**) Schematics of different size effects of active materials on a solution-based LPSCl infiltration process with the same loading value of ~ 17.5 mg/cm^2^. (**b**) FE-SEM images of NCM622-based composite electrodes with different active material sizes (left) 4 µm particles only, (middle) 10 µm particles only, and (right) a mixture of 4 and 10 µm particles.

**Figure 7:**
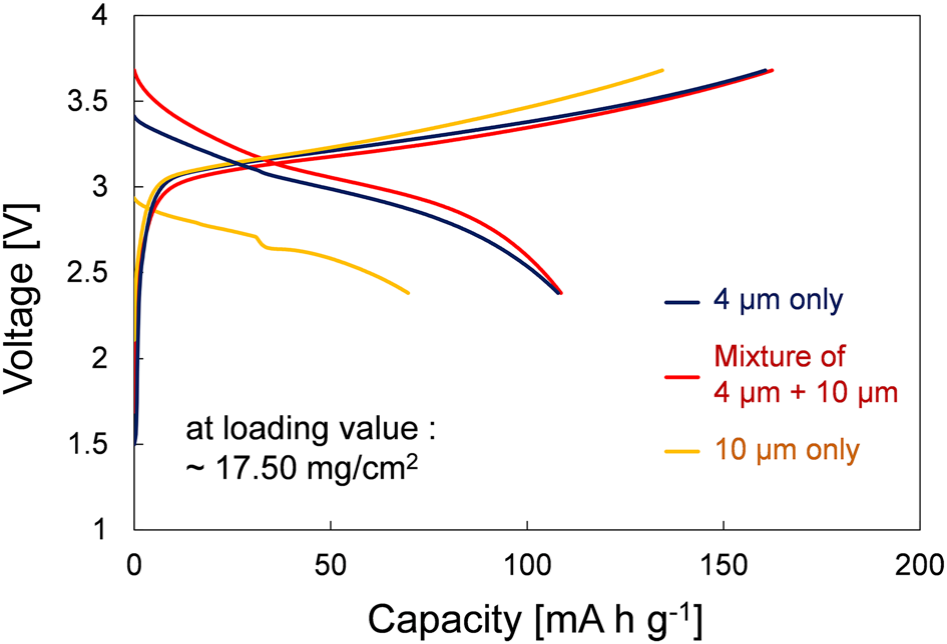
Initial cycle charge and discharge voltage profiles of all-solid-state batteries using the NCM622-based composite electrodes, where those half-cells were fabricated using the LPSCl SE-infiltrated cathodes at loading value of ~ 17.50 mg/cm^2^ using various sizes of active materials: 4 µm particles only (black line), mixture of 4 and 10 µm particles (red line), and 10 µm particles only (yellow line).

For efficient penetration of liquid-phase SEs into the composite electrodes, the particle size of the active materials could also be an important parameter to determine the electrochemical performance of the ASSBs. As shown in Fig. 6, particle size effects of the active materials were investigated using small- (~ 4 µm diameter) and large-size (~ 10 µm diameter) NCM622 particles. It is expected that sheet-type conventional LIB electrodes with large-size particles have large air gaps; hence, the SE solution would be able to penetrate more easily into these porous composite films. However, the electrochemical characteristics of the large-size active materials were poor, with small discharge capacities of ~ 70 mA h g^−1^ at a loading value of 17.5 mg/cm^2^, as shown in Fig. 7. Although the temperatures µm-sized composite electrodes contained a large amount of SEs, their low density had a negative effect on electrode performance. This is attributed to large interfacial resistance and imperfections in the ionic contacts and percolating pathways. Large-size active materials in SE-NCM cathodes can cause severe contact loss at points of contact between active materials and the current collector as well as with LPSCl SEs. Moreover, larger absolute volume changes in the large-size NCM622 particles deteriorate the performance during the charge (de-lithiation) and discharge (lithiation) cycles of ASSBs. In contrast, SE-NCM electrodes that were fabricated using small-sized (4 µm) active materials have lower porosity and higher electrode density than those electrodes made with only 10 µm-sized active materials. However, small particle size may retard the infiltration of SEs due to their high air resistance in micro-pores, thus causing many isolated pores to be left empty, thereby reducing the infiltration rate of SE solutions. In Fig. 7, the initial cycle charge and discharge voltage profiles of only 4 µm-sized active materials exhibit a relatively high capacity of about 105 mA h g^−1^ in the range of 2.5–3.5 V vs Li/Li^+^; their high electrode density is adversely affected by the inefficient and slow infiltration of SE solutions into the small-size active materials in NCM622-based composite electrodes.

Simultaneous enhancement of the electrochemical performance and efficient SE-infiltration process was demonstrated by incorporating a mixture of large-size (10 µm diameter) and small-size (4 µm diameter) active materials in the LIB composite electrodes. In Fig. 6a, a schematic diagram and its corresponding FE-SEM image demonstrate the significantly improved infiltration efficiency of the SEs with different sizes of active materials. It was observed that the mixture of 4 µm and 10 µm active particles reduced the amount of unfavorable spaces inside the electrodes, in comparison to the electrodes fabricated with only 10 µm-sized active materials. This strategy also contributed to the increase in the infiltration rate of the SE solution within the porous electrodes by capillary phenomena, and also helped to obtain intimate contact between electrodes and electrolytes during charge and discharge cycles by mixing large- and small-sized active material particles. In Fig. 7, the initial charge/discharge cycle voltage profile of ASSBs using the SE-NCM electrodes that were prepared with active materials of mixed size showed the best electrochemical performance with a capacity as high as 108.6 mA h g^−1^ in the range of 2.5–3.7 V vs Li/Li^+^, especially at a high loading value of ~ 17.50 mg/cm^2^.

**Figure 8:**
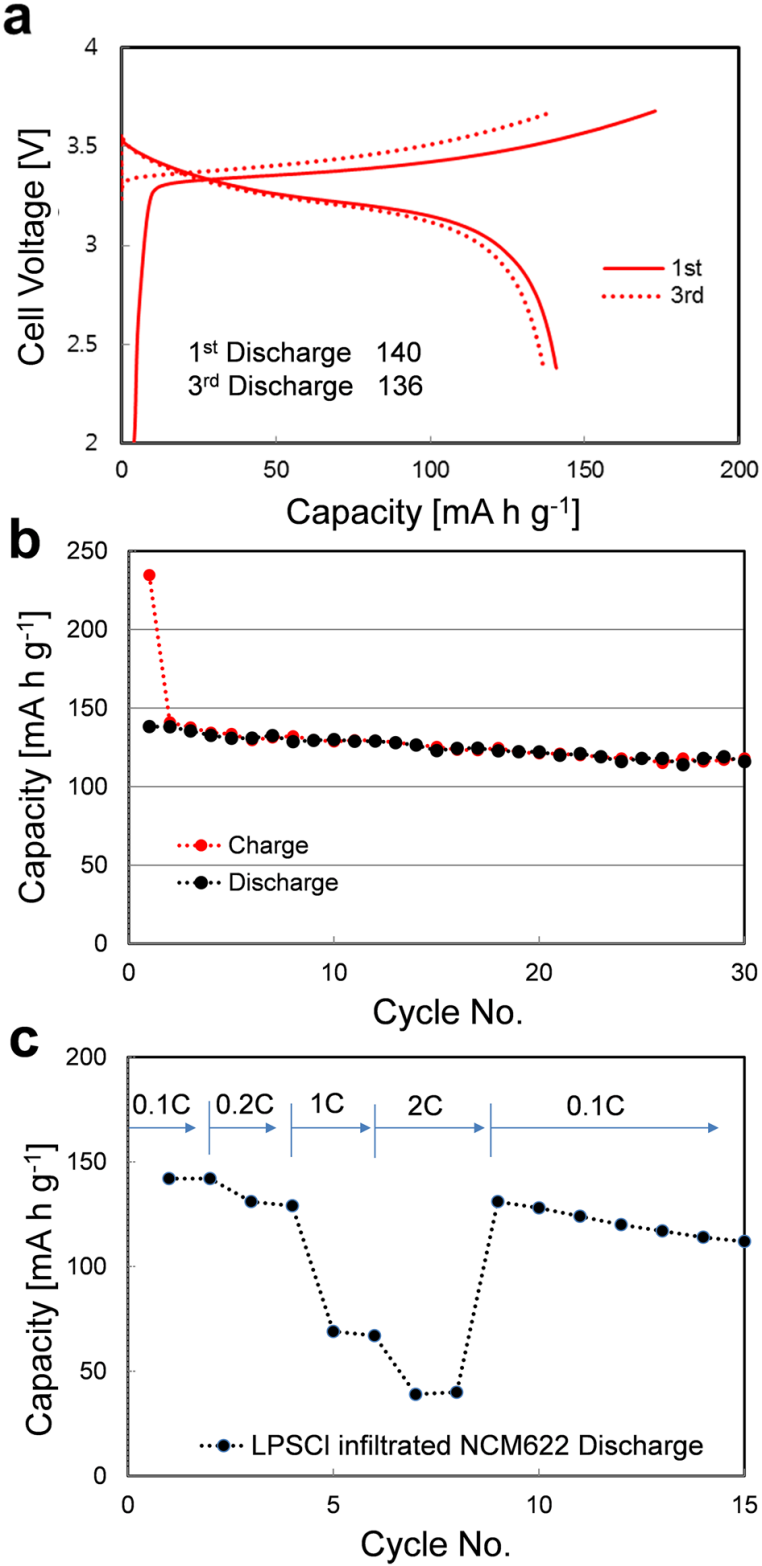
Electrochemical characterization of all-solid-state SE-NCM/Li-In half cells employing the solution-processed LPSCl infiltration technique. (**a**) First and third cycles of charge–discharge voltage profiles at 0.1 C, (**b**) the charge and discharge cycling performance at 0.1 C, and (**c**) C-rate performance at various rates of 0.1 C, 0.2 C, 1 C, and 2 C for all-solid-state batteries with the LPSCl-infiltrated NCM622 and Li_0.5_In electrodes at a loading value of 4.6 mg/cm^2^.

Based on the studies cited above, mixed active material sizes and the solvent vaporization effect were used to fabricate all-solid-state LIB cells employing SE-NCM electrodes via the efficient wet-based infiltration processes. Figure 8 shows the electrochemical characterization of all-solid-state Li_0.5_In/LPSCl-infiltrated-NCM622 cells at a loading value of ~ 4.6 mg/cm^2^ tested under normal operating conditions at 30 °C. Notably, prior to this study, the ASSB cell performance was not available for Li-In ratios of less than 1:4. We found that the Li_0.5_In alloy significantly improved the charge and discharge characteristics when used as a counter electrode. Li and In have molecular weights of 6.94 g/mol and 114.81 g/mol, respectively. Thus, a Li_0.5_In weight ratio of 1:33 wt.% was used as the optimum concentration. When the first and third charge and discharge voltage profiles of all-solid-state LIBs based on Li_0.5_In/LPSCl SE-infiltrated NCM622 (a loading value of 4.6 mg/cm^2^) were measured, the data showed a high initial discharge capacity of 140 mA h g^−1^ at 0.1 C at 30 °C in the voltage range of 2.0–3.6 V vs Li/Li^+^. Moreover, this all-solid-state LIB using the SE-NCM cathode showed stable cycling performance, in which we measured capacity retention as high as 84% at 0.1 C after 30 cycles (Fig. 8b). Figure 8c shows reversible capacities at different current C-rates from 0.1 to 2 C. Promisingly, these values are approaching those of practical LIBs with LEs. Therefore, our solution-based SE infiltration process is expected to be widely utilised for the highly cost-effective and scalable production of high-density and high-capacity ASSBs using sulfide-type SEs. As considering the overall weight of electrodes and SEs, we obtained an energy density of 74.13 Wh/kg_cell_ using the thick electrodes. When we reduce the amount of SE as a membrane, this value can further increase to 209.03 Wh/kg_cell_. Besides, the energy density could be remarkably increased up to 429.59 Wh/kg_cell_ by reducing the thickness of the anode layer, which is similar to the amount of the commercial LIB anode. This cell has a high energy density in comparison with that of conventional LIB cells of 200–300 Wh/kg_cell_. Notably, the high energy density is achievable because it has a high loading value and contains a lower percentage of SE in comparison to the direct mixture of SEs in conventional ASSBs. We believe that our solution-processed SE-infiltration process will effect and provide a higher energy density via optimization of SEs and their processes so that they solve the critical issues of SEs and interfacial resistance within the cathode.

## Conclusion

The efficient infiltration of SE solutions into conventional LIB composite electrodes (NCM622) has been successfully demonstrated and used to fabricate high-capacity ASSBs using high-density and thick cathodes. Increasing the process temperature above the boiling point of the EtOH solvent used in LPSCl SE solutions at 90 °C resulted in a vaporization effect that helped to form intimate interfacial contact between the cathodes and the SE and also ensured a uniform distribution of the SE within the thick electrodes. Therefore, this process enables ASSBs to reach reversible capacities as high as 177 mAh/g at 0.05 C and 90 °C by using LPSCl-infiltrated NCM622 cathodes. This method was also effectively applied to thick electrodes that have high electrode densities (loading values) of ~ 17 mg/cm^2^, resulting in an initial discharge capacity of ~ 136 mAh/g at 0.05 C. Furthermore, the particle size of the active materials in LIB composite electrodes also significantly affected the SE solution infiltration, and therefore the SE content in the electrodes. A mixture of small-sized (4 µm diameter) and large-sized (10 µm diameter) particles induced a high electrode density by filling the pores with small-sized particles as well as sustaining more capillary pores, which leads to strong absorption of the SE solution. The increase in SE content contributes to enhancing the formation of ionic conductive pathways, which results in better ion contact between the SE and the electrodes. The NCM622-based composite electrodes of ASSBs with efficient LPSCl SE-infiltration showed excellent electrochemical performance comparable to that of conventional LIBs using liquid electrolytes. It is expected that engineering of the solvent vaporization effect and mixed particle sizes of active materials during the SE solution infiltration process have great significance that will be important for commercial applications of high-capacity, safe, and scalable ASSBs using sulfide SEs.

## Methods

### Material preparation

NCM622 powder (LiNi_0.6_Co_0.2_Mn_0.2_O_2_, L&F Co. Ltd., South Korea) and PVDF binder (Solvay Corp.) were dried at 80 °C under vacuum for 24 h to completely remove any trapped water. NCM622, Super-P carbon black (carbon additives) and PVDF binder were mixed at a 96:2:2 weight ratio in N-methyl-2-pyrrolidone (NMP) solvent to acquire a viscous slurry followed by stirring at 2000 rpm for 10 min using a Thinky Super Mixer (ARE-310, THINKY Corp.). After mixing the slurry, it was coated onto aluminum (Al) foil and dried in a vacuum oven at 100 °C for 24 h to completely remove all traces of the NMP solvent. The active material loading of the composite cathode was controlled to be ~ 18 mg/cm^2^. To make the LPSCl solution, Li_2_S and P_2_S_5_ were purchased from Sigma-Aldrich Co. Ltd. The weight ratio of Li_2_S/P_2_S_5_/LiCl was fixed at 42.8:41.4:15.8. After adding a fixed amount, the high-energy milling treatment was conducted using a ball milling apparatus. The rotation speed was fixed at 600 rpm for 10 h via an intermittent step. The synthesized LPSCl powder was added to the EtOH solvent at a 0.2:1.578 (wt.%) ratio to prepare the SE solution. The prepared solution was mixed at room temperature for 6 h in an Ar-filled glovebox (Korea Kiyon, dew point under − 80 °C).

### Fabrication of SE-infiltrated electrodes

The as-prepared NCM622-based composite LIB electrodes were infiltrated using a LPSCl SE solution. SE-infiltrated ASSB electrodes were fabricated by dipping the LIB electrode in the SE solution, followed by heat treatment on a hotplate at controlled temperatures of 45, 75, and 90 °C for 10 min. The LIB composite electrodes were then dried at room temperature for 2 h, and at 50 °C for 5 h (all of the above experiments were carried out in an Ar-filled glovebox). Subsequently, prepared samples were heat treated at180 °C for 6 h under high vacuum conditions. Notably, the boiling point of EtOH is 78.3 °C, thus, there should be no EtOH solution left after treatment above 90 °C for more than 10 min. Any remaining EtOH may increase the viscosity of the SE solution and inhibit the infiltration process.

### Characterization

Field-emission scanning electron microscopy (FESEM) and cross-section energy dispersive X-ray spectroscopy (EDS) images of the LPSCl SE-infiltrated NCM622 electrodes were obtained by using a focused ion beam (FIB) (4 kV for 3 h, Ar ion beam) to prepare the samples for measurement using a JSM-7619Plus (JEOL) FESEM. X-ray powder diffraction (XRD) patterns were measured after sealing the samples using a Be window and mounting them on a MiniFlex 600 diffractometer (Riguku Corp.) at 15 mA and 40 kV. Press cells consisting of a Li-In powder anode, Ni-based cathode, and the LPSCl pellet as a membrane were fabricated to evaluate the electrochemical performance of the half-cell test using SE-infiltrated NCM622 composite cathode. The thickness of the LPSCl pellet used for the membrane layer was controlled to be 800 µm. Electrochemical characterization of LPSCl SE-infiltrated NCM622 electrodes was carried out by using all-solid-state NCM622/Li-In powder half-cells at 55 °C. The assembled coin cells were charged and discharged by using a battery cycler (Won-A Tech.) in the potential range of 2.4–3.7 V vs Li/Li^+^.

## Supplementary information

Supplementary Information 1.
