# Supplementary material for: Facile fabrication of solution-processed solid-electrolytes for high-energy-density all-solid-state-batteries by enhanced interfacial contact
Source: Sci Rep. 2020 Jul 17;10:11923. doi: 10.1038/s41598-020-68885-4 (PMC7367834; doi:10.1038/s41598-020-68885-4)
Supplement: Supplementary file 1 — Supplementary Information 1. [file 41598_2020_68885_MOESM1_ESM.docx]

***Supporting Information***

**Facile Fabrication of Solution-Processed Solid-Electrolytes for High-Energy-Density All-Solid-State-Batteries by Enhanced Interfacial Contact**

Min-Ju Kim ^a,b^, Jun-Woo Park ^a,*^, Byung Gon Kim ^a^, You-Jin Lee ^a^, Yoon-Cheol Ha ^a^, Sang-Min Lee ^a^, and Kang-Jun Baeg ^b,**^

^a^ Next Generation Battery Research Center, Korea Electrotechnology Research Institute, Gyeongsangnam-do, 51543, Republic of Korea

^b^ Department of Graphic Arts Information Engineering, Pukyong National University, Busan 48513, Republic of Korea

Correspondence and requests for materials should be addressed to J.-W. Park (e-mail: parkjw@keri.re.kr) K.-J. Baeg (email: kangjun100@pknu.ac.kr)


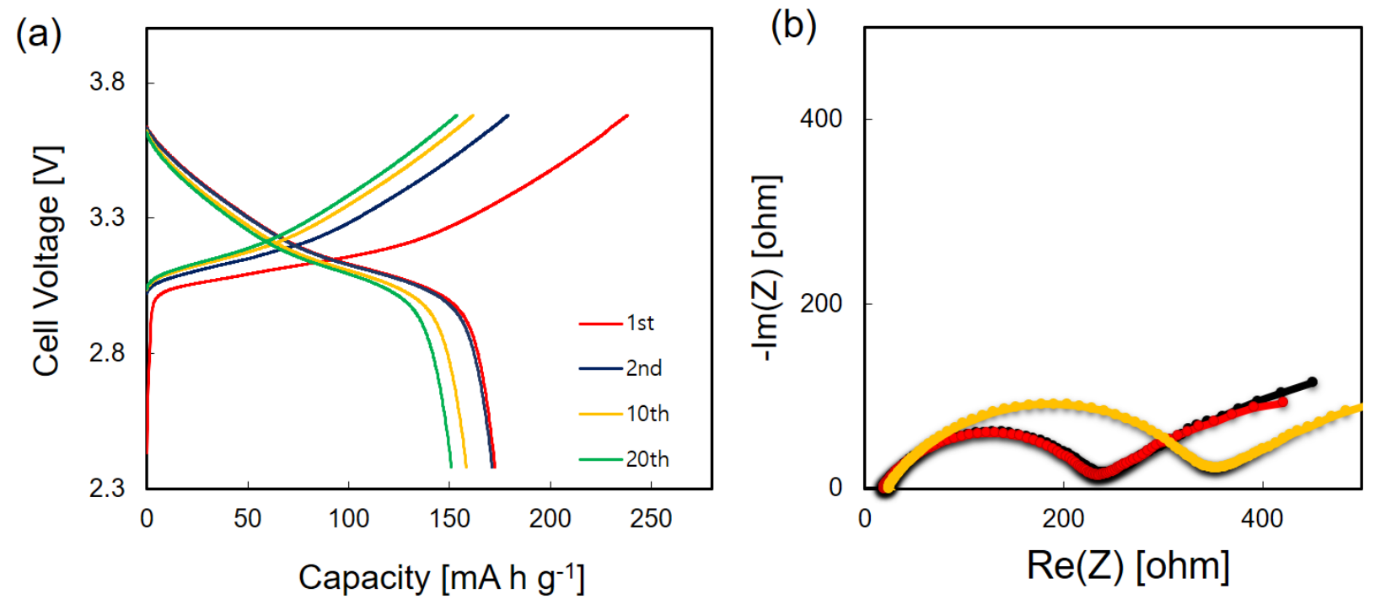


**Figure S1**. (a) Charge and discharge voltage profiles and capacities and (b) Impedance measurement of all-solid-state batteries using the SE-infiltrated NCM622-based composite cathodes during 20^th^ cycling test. Work temperature 55 °C, C-rate 0.05 C, loading value 2.3 mg/cm^2^.


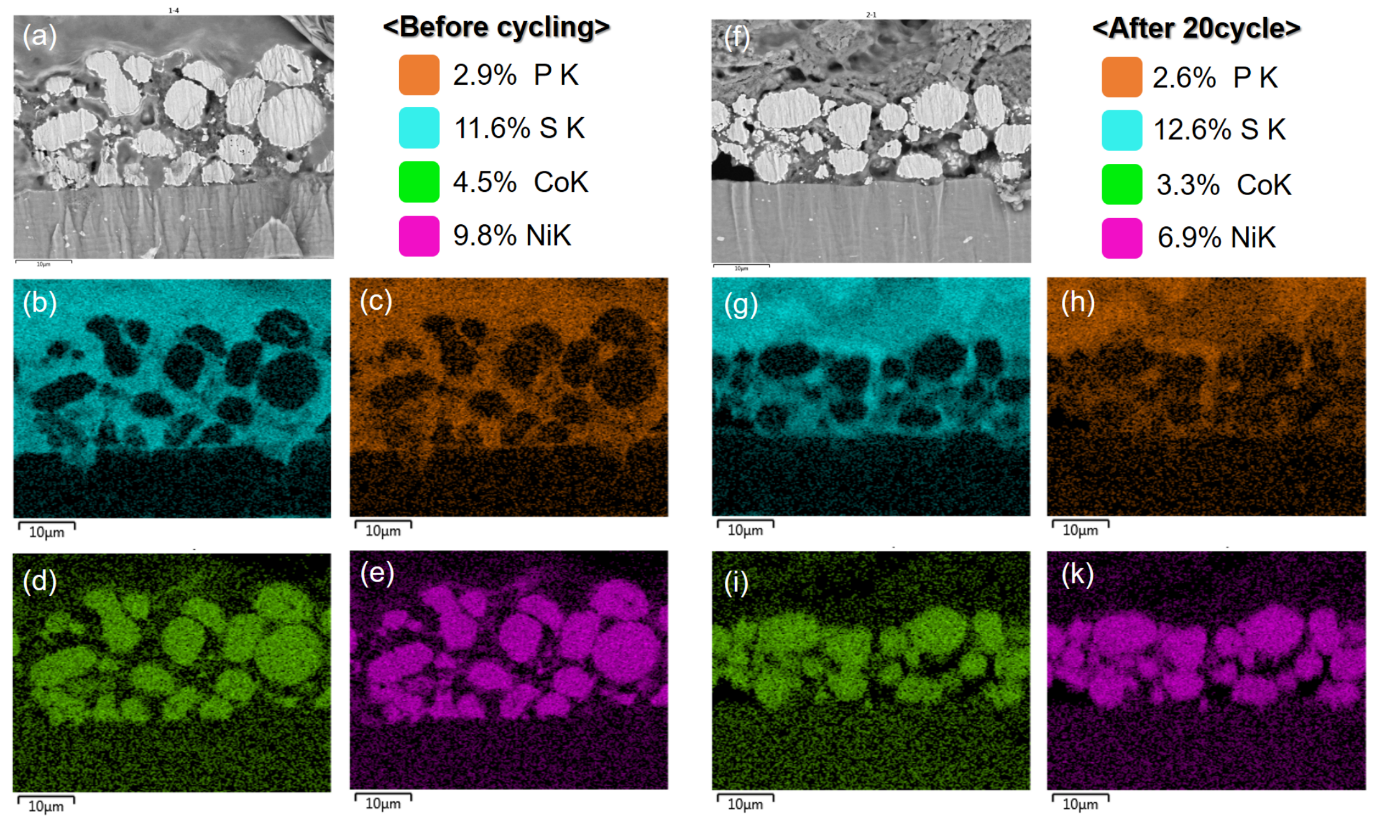


**Figure S2**. Cross-sectional FE-SEM images of LPSCl-infiltrated NCM622-based composite electrodes and their corresponding EDXS elemental maps before and after charge-discharge cycling test. After cutting the electrode cross-section using a CP treatment, the spatial distributions of SE elements (representatively S ions) were analyzed and the uniform distribution of SE inside the electrodes was confirmed when processed at 55 °C.


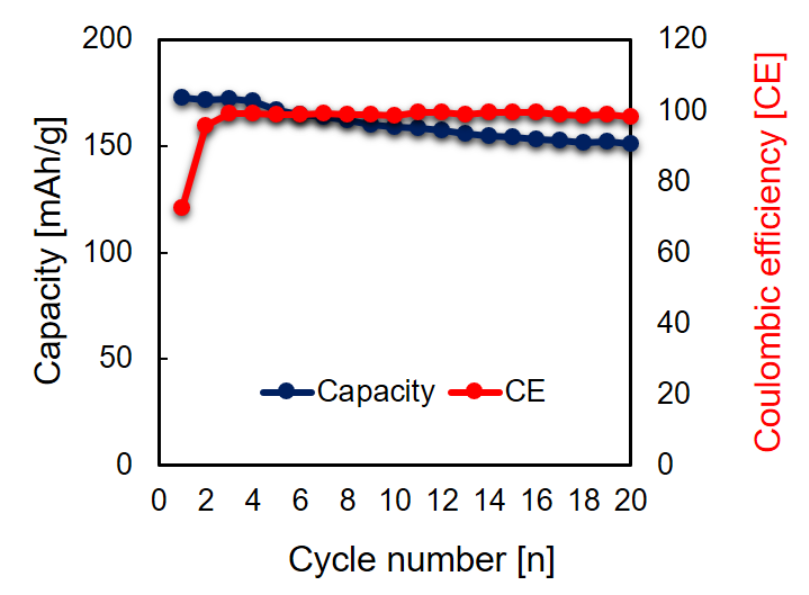


**Figure S3**. Capacity and Columbic efficiency values of the ASSBs using SE-infiltrated cathodes during 20th charge and discharge cycles.


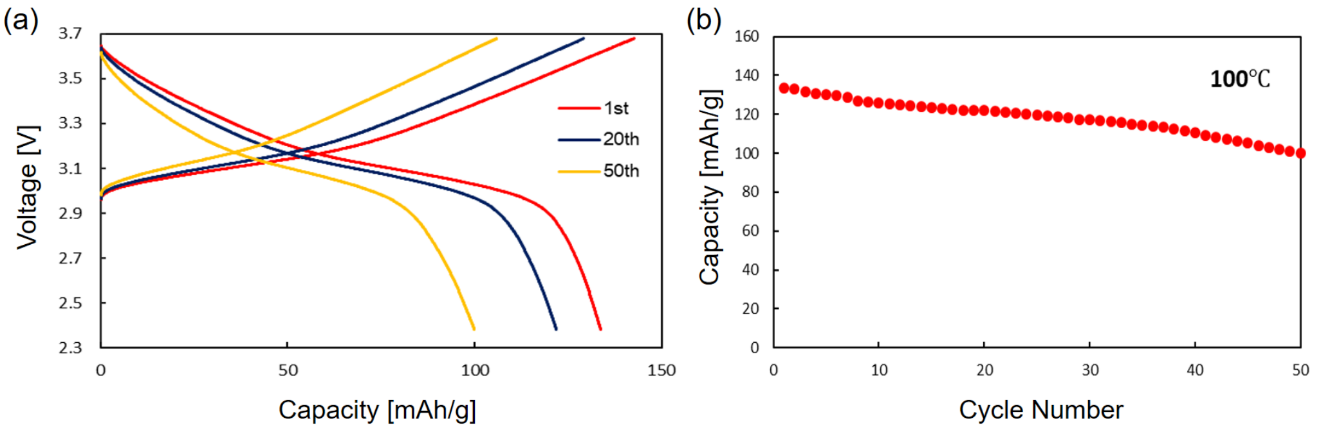


**Figure S4**. Charge and discharge voltage profiles and capacities of ASSBs using the SE-infiltrated NCM622-based composite cathodes during the 50^th^ cycling test. Work temperature 100 °C, C-rate 0.1 C, loading value 15.7 mg/cm^2^.

**Calculation of Energy Density**

As considering the overall weight of electrodes and SEs, we obtained an energy density of 74.13 Wh/kg·cell using the thick electrodes. As can be seen in **Figure S5**, when we reduce the amount of SE as a membrane, this value can further increase to 209.03 Wh/kg_cell_. Besides, the energy density could be remarkably increased up to 429.59 Wh/kg_cell_ by reducing the thickness of the anode layer, which is similar to the amount of the commercial LIB anode. This cell has a high energy density in comparison with that of conventional LIB cells of 200-300 Wh/kg_cell_.

The energy density of ASSB cells was obtained by using the formula, as follows;

𝑚𝐴ℎ/𝑔_cell_ × 3.7𝑉

where *g_cell_* is the weight of cells. **Table S1** shows the various thicknesses of membrane and anode, and their corresponding weights for the calculation of the energy density. At this time, the loading value of the cathode used for the calculation is approximately 17 mg/cm^2^.

^
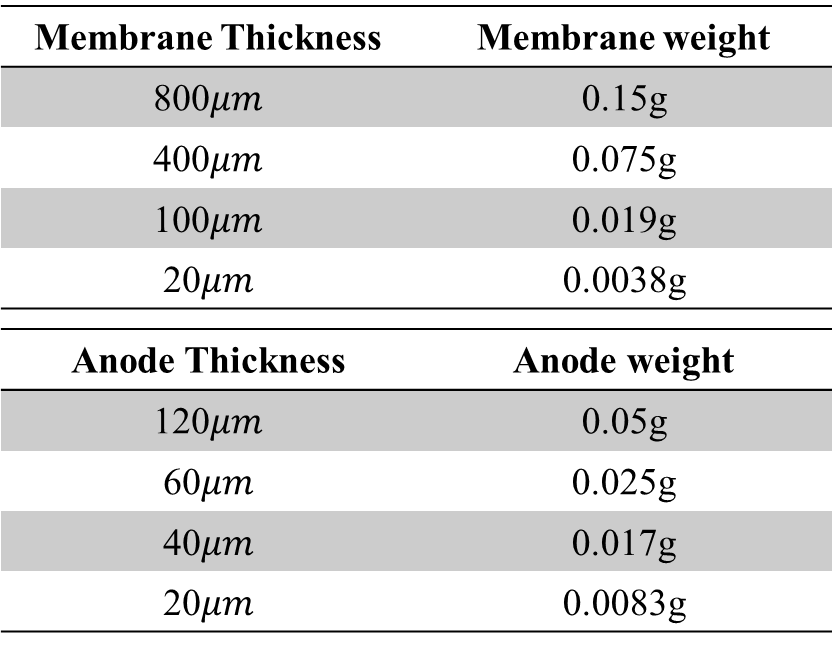
^

**Table S1.** Weight of membrane and anode by the various thickness

**
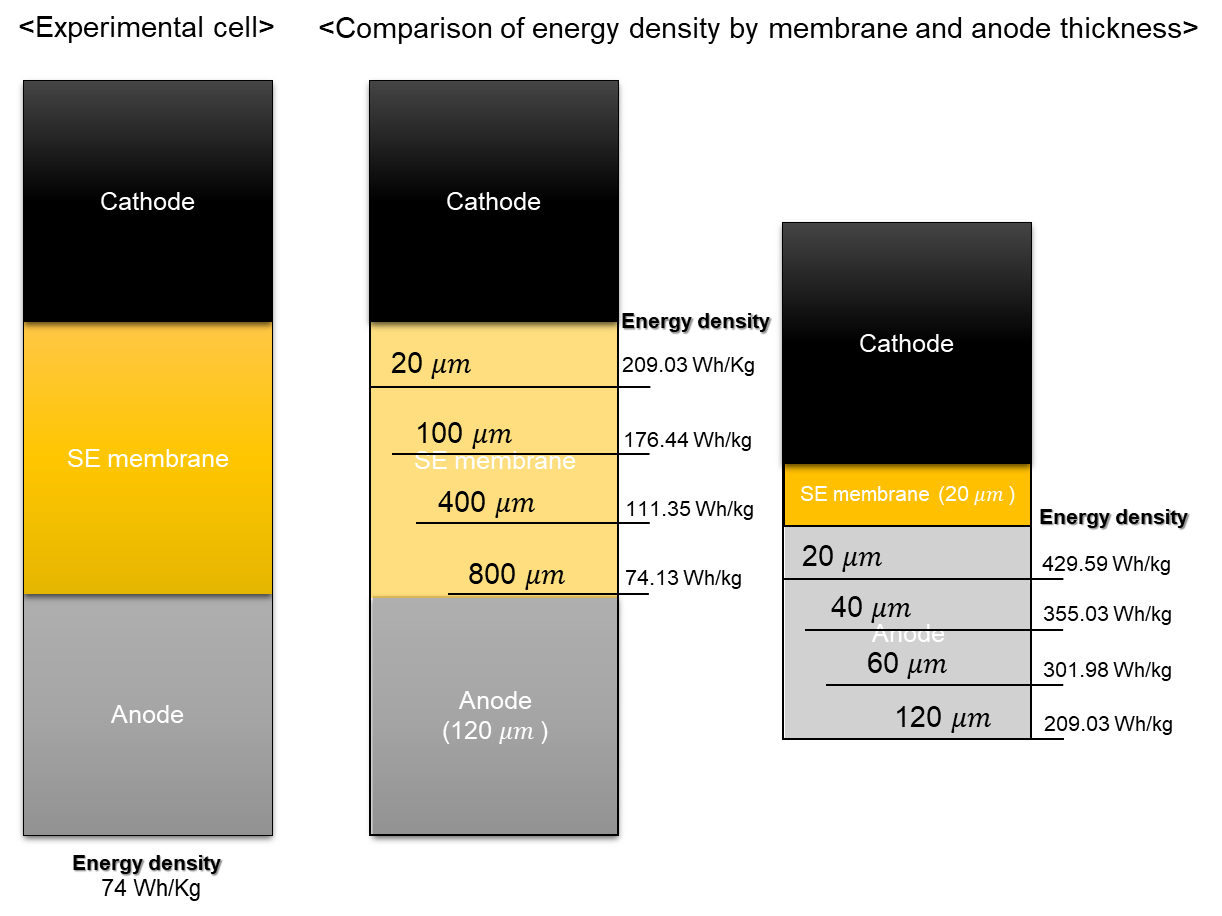
**

**Figure S5**. Comparison of energy density of ASSBs by decreasing the thickness of membrane and anodes.
